# Role of Medical Nutrition Therapy as Treatment of Sarcopenia in Older People with Type 2 Diabetes

**DOI:** 10.3390/nu17010172

**Published:** 2025-01-02

**Authors:** Alessia Gaglio, Valeria Grancini, Federico Giacchetti, Marco Mirani, Emanuela Orsi, Veronica Resi

**Affiliations:** 1 Endocrinology Unit, Fondazione IRCCS Ca’ Granda Ospedale Maggiore Policlinico, Via Francesco Sforza 35, 20122 Milan, Italy; 2 Endocrinology, Diabetology and Andrology Unit, IRCCS Humanitas Research Hospital, Via Manzoni 56, Rozzano, 20089 Milan, Italy; marco.mirani@humanitas.it

**Keywords:** type 2 diabetes, sarcopenia, diet, nutritional status, body composition

## Abstract

Background: Globally, the progressive increase in the aging population has led to social and health problems associated with age-related chronic diseases, such as type 2 diabetes mellitus (T2DM) and sarcopenia. Recent studies have highlighted that sarcopenia and diabetes have a bidirectional relationship. Nutritional therapy is a key element in the treatment of both sarcopenia and diabetes. To date, there are no nutritional guidelines for the management of sarcopenia in T2DM. The aim of this study was to evaluate the efficacy of a muscle-targeted nutritional intervention in older people with sarcopenia and type 2 diabetes based on the Italian nutrition guidelines. Methods: A total of 211 subjects (117 M and 94 F) affected by T2DM with a mean age of 74 ± 6.0 years were screened for sarcopenia, using EWGSOP2 diagnosis criteria, and enrolled to receive personalized dietary plans with two main targets: a daily energy intake of 25–30 kcal/kg body weight and a daily protein intake of at least 1.1–1.2 g/kg body weight. Results: In total, 34 subjects (24 M and 10 F) were sarcopenic with a prevalence of 16%, which was higher in men. After six months of treatment, handgrip strength increased by 0.83 kg (19.57 ± 5.70 kg vs. 20.40 ± 6.10 kg, *p* = 0.649), protein intake improved (0.91 ± 0.28 g/kg body weight vs. 1.03 ± 0.40 g/kg body weight, *p* = 0.115), and the glycated hemoglobin decreased (7.39 ± 0.49% to 6.82 ± 0.98%, *p* = 0.010). Seven younger subjects had an improvement of sarcopenia with a decrease in HbA1c (7.50 ± 0.59% vs. 6.91 ± 0.79, *p* = 0.19). The difference over time in the consumption of saturated fatty acids (OR 0.6, 95% CI 0.33–1.09, *p* = 0.096) and simple sugars (OR 0.91, 95% CI 0.80–1.01, *p* = 0.090) appeared to be associated with an improvement of sarcopenia status. A total of 177 subjects did not meet the criteria for a diagnosis of sarcopenia, and 148 subjects were assessed. The handgrip strength (26.22 ± 9.36 vs. 26.18 ± 9.24 kg, p0.974) and the glycated hemoglobin (7.21 ± 1.07 vs. 7.27 ± 0.98%, *p* = 0.735) remained stable over time, while protein intake at six months increased (0.81 ± 0.29 vs. 0.91 ± 0.29 g/kg body weight, *p* = 0.024). Four people were diagnosed with sarcopenia at follow-up, with a lower handgrip strength test result. These subjects were older and had worse glycemic control (HbA1c + 0.5%). Conclusions: Lifestyle modification is important to prevent or reverse the development of the disease. Nutritional therapy in this population is therefore aimed at meeting all nutritional needs and promoting better glycemic control, in terms of glycated hemoglobin, in order to reduce the development of sarcopenia. Although promising, the intervention requires validation in larger studies with control groups.

## 1. Introduction

Type 2 diabetes mellitus (T2DM) is a metabolic disease characterized by chronic hyperglycemia due to insulin secretion or utilization disorders or both [1]. The global prevalence of diabetes is estimated to be 10.5% (536.6 million people) in 2021, rising to 783.2 million in 2045; the prevalence among 75–79-year-olds was 24% in 2021 and is expected to rise to 24.7% in 2045 [2]. As the world’s population ages, the proportion of people over 60 with diabetes will increase. Sarcopenia is a progressive and generalized disorder of skeletal muscle that commonly occurs with advancing age and is associated with an increased likelihood of a wide range of adverse outcomes, including impaired mobility and increased morbidity and mortality [3,4], and has been reported as an emerging complication in people with diabetes. It is characterized by a gradual loss of skeletal muscle mass and a loss of muscle function [5]. Body composition, especially muscle mass, changes throughout life. Pronounced aged-related changes occur after the 50th life year [6]. This appears to be most pronounced in the seventh decade and beyond. After the age of 70, muscle mass is lost at a rate of 0.5–1.0% per year with a parallel decline in strength of 10% to 15% per decade until the age of 80 years, when the rate of loss accelerates to 25–40% per decade [6,7].

The World Health Organization (WHO) has established a code (International Classification of Diseases and Related Health Problems [ICD-10-CM] [M62.84]) for better diagnosis, assessment, and treatment of this age-related condition [8]. Recently, several definitions of sarcopenia have emerged from different consensus groups (e.g., EWGSOP2, AWGS, SDOC [9]).

Risk factors for sarcopenia include age, gender, level of physical activity, and the presence of chronic diseases such as diabetes. Studies have shown that the risk of sarcopenia in both men and women is 3 times higher in patients with T2DM that in non-diabetic people [10]. Sarcopenia is associated with low glucose disposal at the skeletal muscle site. Skeletal muscle is useful against hyperglycemia, in particular in the post-prandial phase, so preserving skeletal muscle mass prevents the onset of prediabetes and progression to T2DM. In contrast, impaired skeletal muscle glucose uptake due to insulin resistance is amplified by sarcopenia [11].

In epidemiological studies, the prevalence of sarcopenia in T2DM ranges from 7% to 29.3% in different populations [12]. Currently, sarcopenia is considered a new complication of T2DM [13] that not only leads to poor quality of life but also increases the risk of physical disability and even death [14,15].

Sarcopenia has been shown to be a dynamic and reversible process: sarcopenic individuals may improve or worsen their frailty status over the course of their lives. Nutrition is a key element in the pathophysiology of sarcopenia and may play an important role in its prevention and treatment [16]. In diet, low intakes of energy and protein are associated with sarcopenia risk. A mixed promotion of physical activity and nutritional supplementation may also be an effective intervention in sarcopenic patients [17]. There is a relationship between diet quality and sarcopenia: the amount of food and energy intake is important for maintaining muscle mass and physical performance [18,19]. Adequate total protein intake in the usual diet may reduce the risk of sarcopenia [20,21]. An imbalance between protein intake and protein requirements can lead to a loss of skeletal muscle mass. An equal distribution of proteins at each meal (at least three main meals) is preferable to optimize the stimulation of muscle protein synthesis [22]. As sarcopenia is a complex pathology, multimodal interventions appear useful, including exercise, nutritional intervention and other approaches, to prevent or reverse the disease, especially in a pathological condition such as diabetes.

The primary endpoint of this study was to evaluate the efficacy of a muscle-targeted nutritional intervention on muscle strength while the secondary endpoint was to determine the glycemic control and protein intake in a cohort of older people with sarcopenia and type 2 diabetes.

## 2. Materials and Methods

This was a prospective single-center pilot study to evaluate the effectiveness of nutritional intervention in people with sarcopenia. The study was conducted in the diabetology outpatient clinic of the Endocrinology Unit of the Fondazione IRCCS Ca’ Granda Ospedale Maggiore Policlinico in Milano.

Eligible participants were T2DM patients with HbA1c > 48 mmol/mol (>6.5%), people aged ≥ 65 years, BMI ≥ 20 kg/m^2^, who agreed to scheduled follow-up visits. Participants were excluded if they were severely ill, had more than two comorbidities, had plans to migrate from the study area, had HbA1c ≥ 86 mmol/mol (HbA1c ≥ 10%) and BMI ≤ 20 kg/m^2^. The protocol was approved by the local ethics committee and registered with the ClinTrial.gov under number NCT05961878. Study procedures were performed in accordance with the Declaration of Helsinki’s ethical principles for medical research involving human subjects.

### 2.1. Measurements

Sarcopenia was measured according to the guidelines of the European Working Group on Sarcopenia in Older People 2 (EWGSOP2) [5]. Muscle strength was assessed by measuring grip strength using the JAMAR^®^ dynamometer (Patterson Medical, Chicago, IL, USA). Handgrip strength was considered low if the measurement was < 27 kg for men or < 16 kg for women. To confirm the presence of sarcopenia, muscle mass was assessed using the ASMMI (<7.0 kg/m^2^ for men and <5.5 kg/m^2^ for women), an index derived from bioelectric impedance analysis (BIA 101; Akern, Florence, Italy), using Bodygram Plus^®^ v. 1.2.2.8. In addition, muscle function was considered low if the walking speed was ≤0.8 m/s for a 4 m walk.

Anthropometric measurements were taken at baseline and after 26 weeks. Body weight was measured to the nearest 0.1 kg with the study participants in light clothing and barefoot, using a scale. Waist circumference (WC) was measured with a flexible steel tape calibrated in centimeters and graduated in millimeters, with mm graduations directly on the skin at the level of umbilicus. Hip circumference (HC) was assessed at the widest part of the hips. Body mass index (BMI) was calculated using the Quetelet equation (weight (kg)/height (m)^2^) [23].

Fasting blood samples were also collected for various biochemical assays, including glycemia, HbA1c, total cholesterol (TC), HDL cholesterol, triglycerides, transaminases, and creatinine to calculate the GFR (mL/min). All the parameters were measured at baseline and at the end of the six months.

Nutritional behavior and diet were assessed at baseline and at the follow-up with a 3-day food diary to quantify the intake of different nutrients, particularly energy and protein, and an interview was conducted by a nutritionist to obtain a detailed description of the foods and beverages consumed; the portion size was determined by using a photo album of all foods and the preparation was noted (e.g., cooking method, addition of fat, ingredients, etc.). All dietary intake data were analyzed for energy and macro- and micronutrient content using Metadieta^®^ v. Professional 4.0.1. Furthermore, the International Physical Activity Questionnaire (IPAQ) was administered to assess the duration and the quality of physical activity in the seven days prior to the visit at baseline and at six months.

### 2.2. Nutritional Intervention

The nutrition education, counseling, and individual diet plan were applied by trained dieticians. The intervention involved individual nutritional assessments and the prescription of personalized dietary plans with two main targets: a daily energy intake of 25–30 kcal/kg body weight and a daily protein intake of at least 1.1–1.2 g/kg body weight [20] as recommended by national and international guidelines for elderly people. Calories were divided into three meals (and two snacks, if necessary) and verified by 3-day food diaries. In addition, participants were asked about physical activity and the need for weight control. The diet consisted of a total caloric intake of 45–60% from carbohydrate (<10% sugar), 15–20% from protein, and 25–30% from fat (<10% saturated fat, minimal trans-fat intake, and 200 mg/day cholesterol), according to the indications of the Italian Standard of Care 2018 [24] and the Mediterranean diet.

During the patients’ visits, the dietician obtained daily nutrient intake by asking the people to recall the foods and beverages consumed in the past 24 h, in addition to the food diary. The study participants were called twice during the observation period to verify their health status. During calls, through 24 h recall, food advice was reinforced to increase dietary adherence.

### 2.3. Purpose of the Study

The primary outcome of this study was a change in muscle strength in older people with or without sarcopenia and type 2 diabetes over six months. The secondary outcomes were changes in macronutrient intake, biochemical and clinical outcomes such as fasting blood glucose, blood pressure, lipid profile, BMI, and waist circumference. All of these were measured at baseline and endline.

### 2.4. Statistical Analysis

The sample size was based on information from previous studies in sarcopenic elderly female and male subjects that assessed the difference in muscle strength [25]. The sample size of 96 subjects was estimated considering a mean difference of 0.9 kg in handgrip strength, assuming a two-tailed test with alpha set at 0.

Statistical analyses were performed using SPSS 26.0 software (SPSS, Inc., Chicago, IL, USA). Baseline characteristics between the two groups were compared using Student’s two-sample *t*-test for continuous variables and the Chi-squared test for categorical variables. A *p*-value of ≤0.05 indicated statistical significance. The difference between two groups was evaluated using Student’s two-sample *t*-test assuming equal variance. A logistic regression analysis was conducted to explore the variables associated with the development of sarcopenia. Complete case analysis was also performed, excluding 4 participants (3 lost to follow-up and 1 dead) in the sarcopenia group (S group) and a total of 29 participants (11 lost to follow-up and 18 discontinued the study) in the non-sarcopenia group (NS group).

## 3. Results

**Figure 1:**
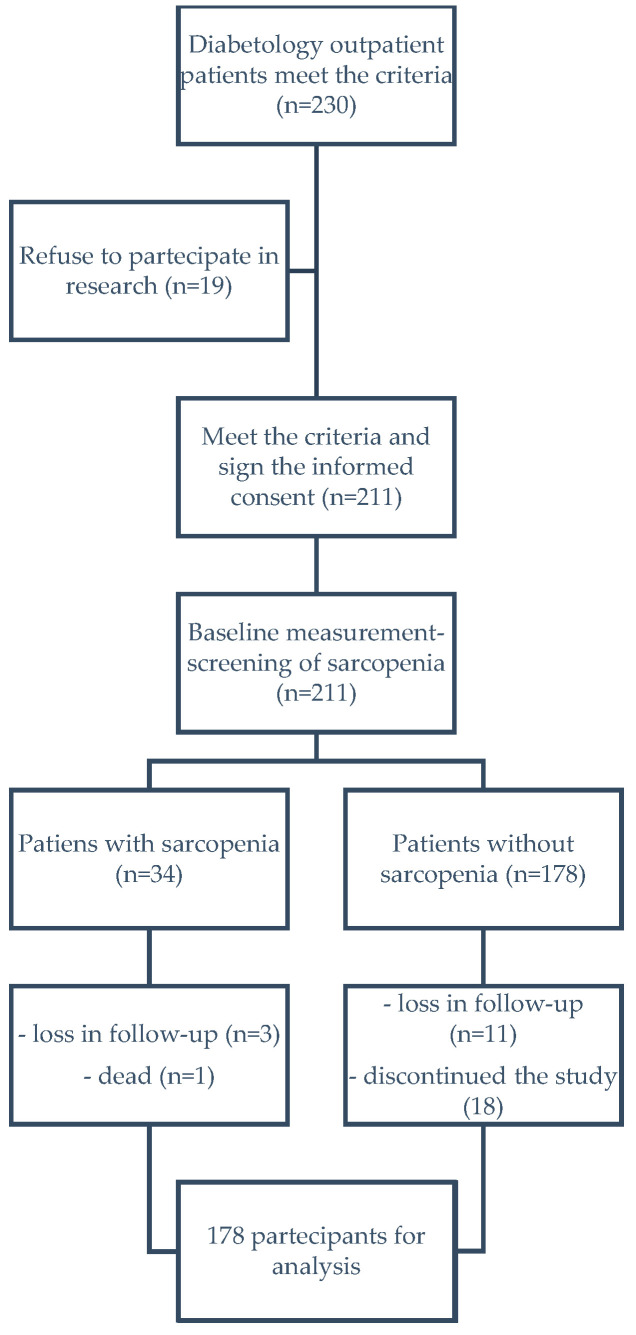
Flowchart of the study.

A total of 211 subjects were recruited consecutively to the nutritional intervention (Table 1). After six months, a total of 178 subjects completed the trial; the remaining 33 people were lost to follow-up. All subjects with type 2 diabetes mellitus were observed at baseline, screened for sarcopenia, educated about the nutritional indications, and re-evaluated after 26 weeks (Figure 1).

The mean age of study participants was 74 ± 6.0 years; 117 subjects were male (M) and 94 were female (F). At baseline, the mean HbA1c level was 7.24 ± 1.0%, and the fasting blood glucose level was 142.32 ± 33.62 mg/dL. Using the EWGSOP2 criteria, 34 subjects (24 M and 10 F) were affected by sarcopenia. The drug therapy had been stable for at least 6 months and did not change during the observation period.

As a sensitivity analysis, we compared the baseline characteristics of the initial 211 subjects with the cohort of subjects (n = 178) who concluded the study. No differences were observed in the mean age (74 ± 6 vs. 74 ± 5 years, *p* = 0.725), diabetes duration (15.5 ± 8.6 vs. 16.6 ± 8.7 years, *p* = 0.324), weight (74 ± 14.85 vs. 74.3 ± 16.1 kg, *p* = 0.89), BMI (27.6 ± 4.9 vs. 27.5 ± 4.7 kg/m^2^, *p* = 0.975), WC (99 ± 12 vs. 99 ± 11 cm, *p* = 0.73), HbA1c (7.2 ± 1 vs. 7.2 ± 1%, *p* = 0.692), eGFR (73 ± 19 vs. 71 ± 20 mL/min, *p* = 0.474), or handgrip (25.1 ± 9 vs. 25.1 ± 9 kg, *p* = 0.978). The homogeneity between the two groups supports the reliability of the subsequent analysis.

The prevalence of sarcopenia in this population was 16%, with a higher prevalence in men. The NS group consisted of 177 subjects (80 M and 68 F). The 34 subjects in the S group had a mean age of 77 ± 7 years and a disease duration of 17 ± 10 years, while the NS group had a mean age of 73 ± 5 years and a disease duration of 15 ± 8 years. The mean age at baseline was statistically significant between the two groups. The analysis showed significant differences in weight, BMI, and waist and hip circumference, which were lower in the S group than in the NS group at T0.

Table 2 and Table 3 show all the investigated parameters (anthropometric parameters, body composition, blood tests, and nutritional information).

A total of 34 subjects received the reported nutritional indications and were assessed at the follow-up. At 6 months, 30 subjects were analyzed. The handgrip value increased by 0.83 kg at 6 months (19.57 ± 5.70 kg vs. 20.40 ± 6.10 kg, *p* = 0.649), without significant differences, and the mean value of the IPAQ score was stable during the follow-up period.

**Figure 2:**
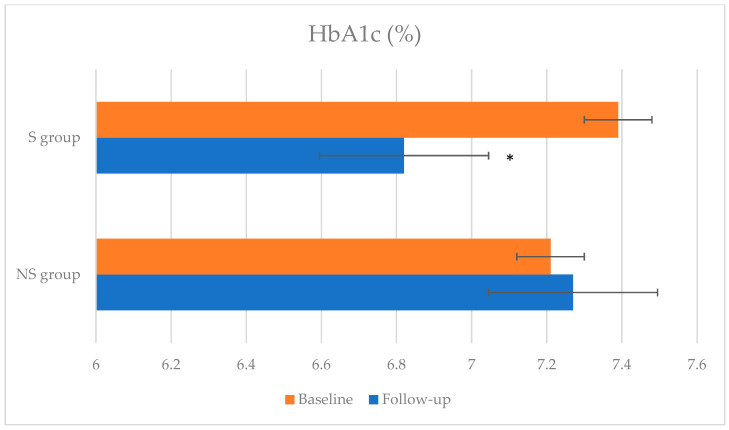
Changes in glycated hemoglobin (%) at baseline and follow-up. * *p* < 0.05.

**Figure 3:**
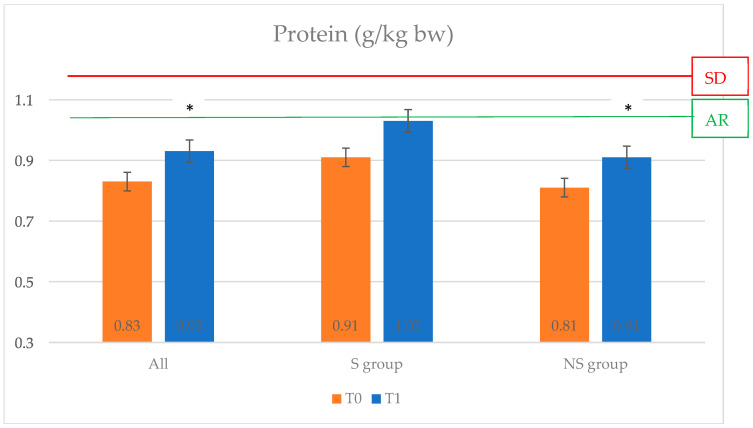
Changes in protein intake (g/kg bw) at baseline and follow-up. * *p* < 0.05.

All the anthropometric parameters and the body composition did not change, whereas the HbA1c decreased from 7.39 ± 0.49% to 6.82 ± 0.98% (*p* = 0.01) (Figure 2). Dietary data showed an increase, but not significant, in protein intake (Figure 3). At baseline, the mean macronutrient intake was 19.25 ± 3.47% of energy from protein, 44.06 ± 7.47% from carbohydrate, and 36.41 ± 8.42% from fat, whereas after 6 months, it was 18.87 ± 3.42% protein, 41.89 ± 6.40% carbohydrate, and 39.08 ± 5.77% fat. Daily fiber intake also increased. In particular, the insoluble fiber intake was statistically significant (7.11 ± 4.67 g vs. 10.51 ± 4.81 g, *p* = 0.022). During the observation period, the cholesterol and the monounsaturated fatty acid intake, especially oleic oil, increased with a statistically significant difference.

During the follow-up, 7 subjects with sarcopenia (4 M and 3 F), with an age of 74 ± 4 years and a disease duration of 19 ± 12 years showed an improvement of sarcopenia status. Interestingly, HbA1c decreased (7.50 ± 0.59% vs. 6.91 ± 0.79, *p* = 0.19), whereas the difference over time in the consumption of saturated fatty acids (OR 0.6, 95% CI 0.33–1.09, *p* = 0.096) and simple sugars (OR 0.91, 95% CI 0.80–1.01, *p* = 0.090) appeared to be associated with remission of sarcopenia.

At baseline, 177 subjects did not meet the criteria for a diagnosis of sarcopenia (NS group). Six months after the nutritional intervention, 148 subjects were assessed (Table 1 and Table 2). The body composition and the BMI and the mean value of handgrip strength remained stable, whereas the IPAQ score seemed to be lower, without a significant difference (913.24 ± 622.84 vs. 835.36 ± 524.85 Met) during the follow-up period.

In the univariable logistic regression analysis, we observed no impact of age (OR 1.057, CI 0.954–1.711, *p* = 0.286), diabetes duration (OR 1.033, CI 0.974–1.095, *p* = 0.281), gender (M/F, OR 0.521, CI 0.165–1.651, *p* = 0.268), HbA1c (OR 0.546, CI 0.247–1.209, *p* = 0.136), eGFR (OR 0.99, CI 0.961–1.021, *p* = 0.534), or protein intake (OR 3.091, CI 0.613–15.575, *p* = 0.171) on sarcopenia development, while BMI (OR 0.747, CI 0.621–899, *p* = 0.002) and handgrip strength at baseline (OR 0.917, CI 0.949–0.991, *p* = 0.029) were associated with a reduced probability of developing sarcopenia.

In a multivariable model considering handgrip, BMI, HbA1c and protein intake, only the first two variables turned out to be independently associated with a lower probability of developing sarcopenia (OR 0.905, CI 0.819–1, *p* = 0.49; OR 0.714, CI 0.555–0.919, *p* = 0.009, respectively).

Protein intake increased as reported in Figure 3. Daily energy intake increased from 1370.97 ± 460.34 kcal at T0 to 1482.54 ± 362.41 Kcal at 6 months, *p* = 0.069. Macronutrient intake (carbohydrates, proteins, and lipids expressed in %) did not change over time. There were significant differences in simple carbohydrates (46.78 ± 19.52 vs. 53.74 ± 23.81, *p* = 0.018) and in saturated fatty acid intake (15.74 ± 6.74 vs. 17.81 ± 8.04 g, *p* = 0.040). The mean daily fiber intake increased: the differences were in the intake of total fiber (16.81 ± 6.47 g vs. 19.39 ± 7.30 g, *p* = 0.007), soluble fiber (2.82 ± 1.21 g vs. 3.25 ± 1.34 g, *p* = 0.015), and insoluble fiber (8.44 ± 5.08 g vs. 9.90 ± 4.95 g, *p* = 0.040). In the NS group, four people were diagnosed with sarcopenia at follow-up, with a lower handgrip strength test. These subjects were older and had worse glycemic control (HbA1c + 0.5%). Furthermore, they had an increased sugar intake, reaching 17.6% of the energy, and their protein intake decreased, without statistical significance.

A total of 211 older people with type 2 diabetes mellitus were observed and 178 were reassessed at 6 months. The mean anthropometric values showed no statistically significant differences. The handgrip value at T0 was 25.15 ± 9.19 kg and remained stable at the follow-up visit (25.18 ± 9.02 kg, *p* = 0.978). At baseline and after the nutritional intervention at six months, protein calorie intake increased significantly (0.83 ± 0.29 vs. 0.93 ± 0.32 g/kg bw, *p* = 0.009), whereas no differences were found in any body parameters and blood tests. Sugar intake (45.53 ± 19.77 vs. 53.33 ± 23.53 g, *p* = 0.011) and total fiber intake (16.73 ± 6.51 vs. 9.09 ± 7.22 g, *p* = 0.006) also increased significantly, as well as soluble fiber (2.76 ± 1.25 vs. 3.20 ± 1.32, *p* = 0.007) and insoluble fiber (8.22 ± 5.03 vs. 10.01 ± 4.90 g *p* = 0.005). In terms of physical activity, assessed by the IPAQ, older people tended to be more sedentary (*p* = 0.039).

## 4. Discussion

This prospective study of older people with type 2 diabetes mellitus allowed us to carry out a careful analysis of the presence and the treatment of sarcopenia. The possibility of an individualized nutritional intervention made it possible to identify the targets of medical nutrition therapy for the management of sarcopenia associated with diabetes.

The prevalence of sarcopenia was 16%, as reported in the literature. The range in elderly people with type 2 diabetes is 7–29% [12], with the highest prevalence in men. A recent meta-analysis by Petermann-Rocha et al. [26] confirms these data using the EWGSOP2 diagnostic criteria, in contrast to what is reported by Cruz-Jentoft et al. [27], which suggests a higher prevalence in women.

In terms of age, a known risk factor for sarcopenia, our population is older than people without sarcopenia. The analysis showed that the subjects with sarcopenia had a lower BMI than the NS group; in fact, the S group was in the normal-weight category, whereas the NS group was in the overweight category. Muscle wasting is associated with weight loss; however, a BMI > 25 kg/m^2^ or BMI > 30 kg/m^2^ could indicate the presence of sarcopenic obesity. According to the diagnostic criteria for sarcopenic obesity in the recent ESPEN-EASO Consensus Statement [28], six subjects in the sarcopenia group had sarcopenic obesity.

Both waist and hip circumferences were smaller in the S group. However, this analysis was not stratified by gender to define the associated cardiovascular risk. Stratification would have resulted in smaller subgroups (24 male, 10 female).

Bioimpedance analysis at baseline revealed differences in cellular water distribution: in the S group, there was a decrease in intracellular water and an increase in extracellular water due to both diabetes, as hyperglycemia draws water to the extracellular level by osmosis [29], and aging, which causes dehydration [30]. Therefore, this result supports the literature and shows that cellular dehydration is associated with reduced muscle function. Furthermore, ICW represents the metabolically active mass (BCM), which was significantly reduced in the S group, proving that BCM and BCMI can be an indicator of sarcopenia, as reported by Rondanelli et al. [31]. BCMI is more sensitive than BMI to changes in muscle mass and protein tissue that may be associated with pathological conditions.

In general, food diaries and 24 h recall analysis showed that adherence to the diet was high on average, but not always consistently followed. The sarcopenia group showed higher compliance on average, probably due to a greater perception of reduced strength and muscle mass. In contrast, the level of physical activity/exercise was very low; in fact, these subjects remained mostly sedentary over time. It can therefore be said that the results obtained are not influenced by lifestyle changes.

The dietary composition of both groups was close to the recommendations for people with diabetes, and the protein intake of all subjects increased (*p* = 0.03) over time; however, both groups had protein intake below the LARN-suggested dietary target (SDT) of 1.1 g/kg of body weight, both at baseline and at follow-up, but protein intakes (g/kg body weight) increased significantly as a result of the nutritional intervention. The NS group, unlike the S group, did not meet the recommended daily intake for the adult population of 0.9 g/kg of body weight. This finding has been widely observed in studies of older people; in fact, the geriatric population consumes less protein for a variety of reasons, including loss of appetite and changes in taste.

The 30 subjects found to be sarcopenic were reassessed after 6 months. The force expressed by the isometric contraction of the handgrip is strongly related to muscle strength. The handgrip value increased by 0.83 kg at 6 months, without significant differences, probably due to the small sample size and a relatively short observation period, but not because of changes in physical activity. However, these data seem important because handgrip strength is a useful indicator of general health, and, in particular, early mortality from all causes and cardiovascular death, as well as disability [32].

The HbA1c test is important for monitoring the efficacy of therapy, as it reflects the average blood glucose levels over the last 3 months. In the S group at follow-up, the mean HbA1c value decreased with a significant difference (*p* = 0.010), improving glycemic control. This result is significant: data in the literature report that higher glycated hemoglobin levels are associated with a higher risk of sarcopenia. Indeed, numerous studies confirm a higher prevalence of sarcopenia in people with type 2 diabetes mellitus, with higher glycated hemoglobin levels and a longer duration of the disease [33]. A reduction in the mean HbA1c value was achieved in this population without any change in therapy during follow-up and without any episodes of hypoglycemia. This last aspect is important as the population observed is elderly.

Analysis of food diaries after the dietary intervention shows a non-significant increase in daily protein intake, approaching the SDT of 1.1 g/kg of body weight. The failure to achieve the desired target could be due to the small number of subjects in the S group, but adequate energy and protein intake can help prevent loss of muscle mass. The amount of carbohydrate intake was less than 45% of the energy, with an increase in the intake of simple carbohydrates. There was a significant increase in the consumption of insoluble fiber, probably due to the choice of wholemeal cereals, as recommended by the dietary guidelines. The average cholesterol intake significantly increased, but remained within the recommended range (<300 mg), as did the consumption of monounsaturated fatty acids, oleic acid, and saturated fatty acids. The subjects were advised to use EVO oil as a seasoning. The literature suggests that EVO oil can potentially improve muscle structure and function and has anti-inflammatory properties [34].

After 6 months, seven subjects showed an improvement in sarcopenia status, their average handgrip value increased without significance, and they were younger than the S group with lower HbA1c levels. Protein intake did not increase, despite the recommendations, but they showed an increase in plant-based proteins, and data from the literature showed that protein derived from plants may be the most appropriate source to ensure protein needs in older adults with T2DM [35]. Public health interventions targeting this population may be needed to encourage cooking and plant-based protein consumption. However, despite the evidence that protein intake is crucial for stimulating muscle growth, the long-term evidence on dietary protein intake and muscle mass without resistance exercise is less consistent. During the follow-up, there was a decrease in sugar intake and an increase in carbohydrate intake, without significant differences. In fact, sugar intake decreased from 15.7% to 13.3%. Reducing sugar intake is fundamental for diabetes management, as it helps to prevent an increase in glycemic variability and to achieve post-prandial glycemic values within the desired target [24]. Moreover, a decrease in saturated fatty acids and an increase in monounsaturated fatty acid intake were observed. The difference between the two times of consumption of simple sugars and also saturated fatty acids by these seven subjects seems to be a predictive factor for the remission of sarcopenia, although without significant differences due to the small sample size. In conclusion, the analysis of the data shows that younger subjects with sarcopenia improve their handgrip strength and the state of sarcopenia if they are treated with a personalized nutritional intervention with reduced sugar intake, even if they do not achieve adequate protein consumption.

At follow-up, the 148 subjects in the NS group were overweight and there was no deterioration in handgrip strength over time, probably due to the diet.

Despite the nutritional intervention, there was an increase in daily calorie intake, although not significant, which may help to maintain weight. Although the target of 1.1 g/kg of body weight per day was not reached, the daily protein intake (g/kg of body weight) increased. This shows that the nutritional intervention allows an improvement in the diet in terms of protein intake, which is useful for preventing the development of sarcopenia and improving the body composition of geriatric subjects. These data were not observed in subjects with sarcopenia due to the small sample size. The saturated fatty acids increased, remaining around 10% of the daily energy intake. An adequate intake of saturated fatty acids prevents worsening of the lipid profile.

Daily fiber intake, both total and soluble/insoluble, increased; indeed, the importance of eating more whole grains, legumes, and vegetables was emphasized during the nutrition meeting. However, the mean value of 19.39 ± 7.30 g of total dietary fiber remains below the 25 g/day recommended for the healthy population. It is possible that in order to reach the recommended levels, a longer period of time and other meetings, even intermediate ones, will be needed to reinforce the nutritional information. The dietary intervention favored an increase in protein consumption, which is necessary for the elderly and people with diabetes to maintain a non-sarcopenia state.

Within the NS group, four subjects were diagnosed with sarcopenia at follow-up, with a lower handgrip strength test result. These subjects were older and had worse glycemic control (HbA1c + 0.5%), in contrast to seven subjects who were in remission. Higher glycated hemoglobin levels are known to be associated with poorer nutritional status, a higher incidence of sarcopenia, and reduced muscle mass [36]. The food diaries showed an increase in the percentage of sugar intake, reaching 17.6% of the energy, a value above the target for people with diabetes. The mean values for protein intake were reduced after six months, although there were no significant differences. This contributed to the worsening of the subjects’ nutritional status, leading to the diagnosis of sarcopenia. Patients in this group appeared to be more sedentary. These data seem to be confirmed by the analysis of the IPAQ. Physical activity, especially resistance exercise, combined with an adequate protein intake, seems to be protective against sarcopenia and may be useful for improving muscular health such as strength, mass, and performance [37].

Therefore, nutritional therapy is important to ensure adequate nutrient intake, in particular protein intake, and to achieve optimal glycemic control in elderly individuals with type 2 diabetes, in order to improve the metabolic status of these individuals. MNT, based on the Mediterranean diet, is easily applicable in clinical practice.

The main limitations of the study are the lack of a control group and the small sample size, especially in the sarcopenia group, which does not allow stratification by gender, and the short follow-up. Studies with a control group are needed to assess possible differences in different populations. In addition, further interventional studies are needed to improve the knowledge of the effects of dietary quality on sarcopenia and its components in people with T2DM. Studies of the comparative effects of individual and combinations of different types of physical activity and dietary patterns are needed to refine preventive and therapeutic strategies.

## 5. Conclusions

Sarcopenia is a progressive and generalized condition of age-related loss of muscle mass and strength. It is associated with an increased risk of falls and fractures, leading to a decline in physical function and quality of life. In addition, diabetes can increase the risk and worsen the condition. It is therefore important to be able to detect sarcopenia early, especially in older people with diabetes, in order to improve treatment. In particular, this study looked at the role of nutritional therapy on sarcopenia and lifestyle factors such as diet and physical activity in these people.

Age is one of the non-modifiable parameters for the development of sarcopenia, while lifestyle improvements are important to prevent or reverse the disease. In particular, adequate nutritional therapy, in terms of micro- and macronutrients for older people, appears to be useful, but not sufficient to improve the health status of diabetic people over 65 who have other frailties, such as sarcopenia. Nutritional therapy in this population should therefore aim to meet all nutritional needs and, with pharmacological therapy, achieve better glycemic control in order to reduce the development of sarcopenia. At the same time, physical activity should be encouraged to enable muscle protein synthesis and limit its loss over time. Therefore, specific exercise methods and protein-rich nutrition combination-specific plans should be adopted, and appropriate hypoglycemic drugs selected on an individual basis.

## Figures and Tables

**Table 1:** Baseline characteristics.

Variables	All (N = 211)	Male (N = 117)	Female (N = 94)
**Age (years)**	7.91 ± 5.46	73.91 ± 5.74	73.93 ± 5.10
**Time with diabetes (years)**	15.50 ± 8.63	15.20 ± 8.36	15.90 ± 8.94
**Weight (kg)**	74.06 ± 14.85	78.48 ± 14.48	68.57 ± 13.40
**BMI (kg/m^2^)**	27.61 ± 4.90	27.51 ± 4.58	27.73 ± 5.27
**WC (cm)**	99.63 ± 12.20	101.06 ± 12.02	97.88 ± 12.20
**HC (cm)**	102.79 ± 10.93	101.81 ± 9.11	103.99 ± 12.70
**Handgrip (kg)**	25.15 ± 9.17	30.70 ± 7.67	18.25 ± 5.44
**ASMMI (kg/m^2^)**	6.92 ± 1.12	7.38 ± 1.03	6.35 ± 0.95
**TBW (%)**	52.39 ± 7.45	55.27 ± 6.88	48.86 ± 6.56
**ECW (%)**	51.72 ± 7.61	51.19 ± 8.06	52.37 ± 6.96
**ICW (%)**	48.17 ± 7.85	48.89 ± 8.00	47.27 ± 7.57
**BCM (%)**	47.54 ± 7.20	48.17 ± 7.25	46.78 ± 7.07
**BCMI**	9.57 ± 2.99	10.48 ± 3.46	8.48 ± 1.78
**Glycemia (mg/dL)**	142.32 ± 33.3	146.70 ± 30.23	137.11 ± 36.41
**HbA1c (%)**	7.25 ± 1.00	7.19 ± 0.74	7.32 ± 1.25
**Total cholesterol (mg/dL)**	162.83 ± 39.37	151.26 ± 35.16	177.29 ± 39.57
**HDL cholesterol (mg/dL)**	50.07 ± 13.70	47.19 ± 13.43	53.48 ± 13.23
**LDL cholesterol (mg/dL)**	87.50 ± 32.40	80.98 ± 30.32	94.96 ± 33.09
**Triglycerides (mg/dL)**	137.65 ± 72.89	123.41 ± 63.10	153.63 ± 79.56
**AST (u/L)**	21.22 ± 7.52	22.34 ± 8.99	19.94 ± 5.06
**ALT (u/L)**	22.32 ± 12.57	24.84 ± 14.98	19.42 ± 8.10
**Creatinine (mg/dL)**	0.98 ± 0.37	1.07 ± 0.42	0.86 ± 0.25
**GFR (mL/min)**	73.00 ± 18.84	72.83 ± 17.83	73.20 ± 20.00
**Protein (g/kg)**	0.83 ± 0.29	0.81 ± 0.26	0.85 ± 0.31
**Protein (%)**	18.66 ± 5.62	18.71 ± 4.10	18.59 ± 7.06
**Carbohydrates (%)**	42.96 ± 8.63	42.77 ± 8.66	43.18 ± 8.59
**Fat (%)**	38.27 ± 8.51	38.41 ± 9.09	38.09 ± 7.74
**Energy (kcal)**	1360.73 ± 445.38	1402.78 ± 437.33	1308.28 ± 449.74
**Starch (g)**	90.93 ± 35.02	95.91 ± 38.11	84.78 ± 29.64
**Sugar (g)**	46.53 ± 19.72	46.29 ± 18.90	46.84 ± 20.68
**Fiber tot/1000 kcal**	12.62 ± 3.66	12.53 ± 3.68	12.73 ± 3.63
**Total fiber (g)**	16.73 ± 6.50	17.28 ± 7.11	16.05 ± 5.57
**Soluble fiber (g)**	2.76 ± 1.24	2.83 ± 1.29	2.68 ± 1.18
**Insoluble fiber (g)**	8.22 ± 5.01	8.61 ± 5.70	7.74 ± 3.97
**Cholesterol (mg)**	153.20 ± 76.18	159.62 ± 74.34	144.23 ± 78.06
**Saturated fatty acids (g)**	15.53 ± 6.66	15.97 ± 7.21	14.97 ± 5.87
**Monounsaturated fatty acids (g)**	28.00 ± 14.37	28.97 ± 17.26	26.79 ± 9.55
**Oleic acid (g)**	26.71 ± 14.07	27.68 ± 17.26	25.51 ± 9.29
**Polyunsaturated fatty acids (g)**	6.77 ± 4.69	7.31 ± 5.91	6.10 ± 2.28
**IPAQ (met)**	870.72 ± 4.69	1097.91 ± 981.30	580.42 ± 609.04

**Table 2:** Changes in anthropometric and biochemical parameters from baseline to six months.

Variables	Sarcopenia Group Mean (SD) (N = 34)	*p*-Value	Non-Sarcopenia Group Mean (SD) (N = 177)	*p*-Value
**Weight (kg)**				
at baseline	65.45 (10.15)	0.976	75.71 (15.10)	0.790
at six months	65.40 (9.40)		76.29 (5.14)	
**BMI (kg/m^2^)**				
at baseline	23.90 (2.90)	0.946	28.32 (4.91)	0.925
at six months	23.96 (3.16)		28.38 (4.69)	
**WC (cm)**				
at baseline	91.91 (8.54)	0.764	101.05 (12.30)	0.749
at six months	91.00 (8.65)		100.48 (11.62)	
**HC (cm)**				
at baseline	96.28 (6.45)	0.930	103.98 (11.19)	0.433
at six months	96.09 (5.24)		105.35 (11.65)	
**Handgrip (kg)**				
at baseline	19.57 (5.70)	0.649	26.22 (9.36)	0.974
at six months	20.40 (6.10)		26.18 (9.24)	
**ASMMI (kg/m^2^)**				
at baseline	6.11 (0.82)	0.637	7.08 (1.10)	0.926
at six months	6.24 (0.97)		7.09 (1.10)	
**TBW (%)**				
at baseline	53.42 (10.32)	0.407	52.18 (6.80)	0.669
at six months	55.77 (4.82)		52.60 (6.07)	
**ECW (%)**				
at baseline	57.04 (11.15)	0.854	50.67 (6.27)	0.325
at six months	57.68 (11.07)		51.70 (8.89)	
**ICW (%)**				
at baseline	42.97 (11.12)	0.852	49.17 (6.65)	0.456
at six months	42.33 (11.05)		48.37 (8.82)	
**BCM (%)**				
at baseline	42.85 (9.33)	0.943	48.45 (6.37)	0.354
at six months	42.63 (11.87)		47.46 (9.50)	
**BCMI**				
at baseline	7.89 (1.92)	0.313	9.89 (3.05)	0.820
at six months	9.51 (1.69)		9.79 (2.52)	
**Glycemia (mg/dL)**				
at baseline	134.17 (24.81)	0.724	143.81 (34.84)	0.391
at six months	137.15 (26.28)		139.68 (26.80)	
**HbA1c (%)**				
at baseline	7.39 (0.49)	0.010	7.21 (1.07)	0.735
at six months	6.82 (0.98)		7.27 (0.98)	
**Total cholesterol (mg/dL)**				
at baseline	160.07 (45.24)	0.958	163.35 (38.47)	0.254
at six months	159.31 (37.31)		156.98 (32.82)	
**HDL** **cholesterol (mg/dL)**				
at baseline	50.15 (16.49)	0.803	50.06 (13.25)	0.205
at six months	51.46 (12.48)		47.56 (12.37)	
**LDL** **cholesterol (mg/dL)**				
at baseline	88.12 (33.16)	0.936	87.38 (32.50)	0.656
at six months	89.00 (28.38)		85.25 (27.77)	
**Triglycerides (mg/dL)**				
at baseline	110.35 (56.84)	0.697	142.62 (74.80)	0.641
at six months	102.77 (56.00)		137.15 (81.93)	
**AST (u/L)**				
at baseline	21.27 (11.66)	0.777	21.22 (6.70)	0.184
at six months	22.63 (7.39)		23.53 (9.74)	
**ALT (u/L)**				
at baseline	21.67 (16.96)	0.647	22.45 (11.88)	0.778
at six months	18.38 (11.84)		20.14 (9.77)	
**Creatinine (mg/dL)**				
at baseline	0.86 (0.23)	0.077	0.99 (0.39)	0.685
at six months	1.03 (0.29)		1.02 (0.46)	
**GFR (mL/min)**				
at baseline	78.31 (16.43)	0.105	72.07 (19.19)	0.901
at six months	67.50 (22.04)		71.70 (20.50)	

**Table 3:** Changes in nutritional and lifestyle parameters from baseline to six months.

Variables	Sarcopenia Group Mean (SD) (N = 34)	*p*-Value	Non-Sarcopenia Group Mean (SD) (N = 177)	*p*-Value
**Protein (g/kg)**				
at baseline	0.91 (0.28)		0.81 (0.29)	
at six months	1.03 (0.40)	0.115	0.91 (0.29)	0.024
**Protein (%)**				
at baseline	19.25 (3.47)		18.55 (5.96)	
at six months	18.87 (3.42)	0.721	18.31 (3.28)	0.746
**Carbohydrates (%)**				
at baseline	44.06 (7.78)		42.74 (8.81)	
at six months	41.89 (6.40)	0.337	43.26 (8.19)	0.672
**Fat (%)**				
at baseline	36.41 (8.42)		38.63 (8.53)	
at six months	39.08 (5.77)	0.258	38.77 (7.20)	0.900
**Energy (kcal)**				
at baseline	1258.76 (402.42)		1370.97 (460.34)	
at six months	1384.38 (340.59)	0.286	1482.54 (362.41)	0.069
**Starch (g)**				
at baseline	87.09 (36.02)		91.67 (34.99)	
at six months	90.72 (28.98)	0.726	96.10 (33.27)	0.358
**Sugar (g)**				
at baseline	45.26 (21.29)		46.78 (19.52)	
at six months	51.49 (22.83)	0.351	53.74 (23.81)	0.018
**Fiber tot/1000 kcal**				
at baseline	13.16 (4.21)		12.51 (3.56)	
at six months	14.19 (4.30)	0.432	13.16 (4.21)	0.186
**Total fiber (g)**				
at baseline	16.33 (6.82)		16.81 (6.47)	
at six months	18.78 (6.20)	0.228	19.39 (7.30)	0.007
**Soluble fiber (g)**				
at baseline	2.44 (1.38)		2.82 (1.21)	
at six months	3.02 (1.23)	0.167	3.25 (1.34)	0.015
**Insoluble fiber (g)**				
at baseline	7.11 (4.67)	0.022	8.44 (5.08)	0.040
at six months	10.51 (1.23)		9.90 (4.95)	
**Cholesterol (mg)**				
at baseline	133.10 (59.32)	0.012	169.80 (62.11)	
at six months	187.96 (88.28)		168.01 (108.22)	0.943
**Saturated fatty acids (g)**				
at baseline	14.40 (6.31)	0.297	15.74 (6.74)	0.040
at six months	16.43 (6.48)		17.81 (8.04)	
**Monounsaturated fatty acids (g)**				
at baseline	23.11 (10.11)		28.94 (14.93)	
at six months	29.18 (7.44)	0.037	30.95 (9.64)	0.294
**Oleic acid (g)**				
at baseline	21.84 (9.84)		27.65 (14.62)	
at six months	27.91 (7.07)	0.032	29.40 (9.16)	0.350
**Polyunsaturated fatty acids (g)**				
at baseline	5.89 (2.69)		6.94 (4.98)	
at six months	6.48 (1.93)	0.436	7.16 (4.63)	0.747
**IPAQ (met)**				
at baseline	649.09 (561.33)		913.24 (622.84)	
at six months	640.89 (341.33)	0.251	835.36 (524.85)	0.223

## Data Availability

The original contributions presented in this study are included in the article. Further inquiries can be directed to the corresponding author.
